# Ocular delivery of Pigment Epithelium-Derived Factor (PEDF) as a neuroprotectant for Geographic Atrophy

**DOI:** 10.14336/AD.2024.0216-1

**Published:** 2024-10-01

**Authors:** Emily F. Warner, Laura Vaux, Kara Boyd, Peter S. Widdowson, Katie M. Binley, Andrew Osborne

**Affiliations:** Ikarovec Limited., The Norwich Research Park Innovation Centre, Norwich, NR4 7GJ, United Kingdom

**Keywords:** Geographic atrophy, Neuroprotectant, Pigment Epithelium-Derived Factor, Age-related macular degeneration

## Abstract

Geographic atrophy (GA) is an advanced form of age-related macular degeneration (AMD), that starts with atrophic lesions in the outer retina that expand to cover the macula and fovea, leading to severe vision loss over time. Pigment Epithelium-Derived Factor (PEDF) has a diverse-range of properties, including its ability to promote cell survival, reduce inflammation, inhibit angiogenesis, combat oxidative stress, regulate autophagy, and stimulate anti-apoptotic pathways, making it a promising therapeutic candidate for GA. However, the relatively short half-life of PEDF protein has precluded its potential as a clinical therapy for GA since it would require frequent injections. Therefore, we describe administration of a PEDF gene, comparing and contrasting delivery routes, viral and non-viral vectors, and consider the critical challenges for PEDF as a neuroprotectant for GA.

## Introduction

Geographic Atrophy (GA) is an advanced form of age-related macular degeneration (AMD) characterized by the progressive degeneration of the retinal pigment epithelium (RPE) and photoreceptor cells in the macula. It is a leading cause of severe vision loss and currently lacks effective treatment options. Pigment Epithelium-Derived Factor (PEDF) is a multifunctional neuroprotective protein that maintains RPE and photoreceptor homeostasis, however, its potential to be mobilised in the treatment of GA has yet to be explored clinically. The protective role of endogenous PEDF in preclinical models of neurodegeneration has recently been reviewed by Polato and Becerra [1]. Herein we describe the key hallmarks of GA and how the genetic delivery of PEDF could be a promising therapeutic candidate as a neuroprotectant in the treatment of GA.

### PEDF and Neuroprotection in Geographic Atrophy

The pathogenesis of GA starts with the accumulation of focal deposits of extracellular debris called drusen between the RPE and Bruch’s membrane, which occur as a consequence of aging and are visible in early-stage “dry”-AMD, leading to increased oxidative stress, inflammation, and a cascade of destructive processes. In healthy eyes, RPE cells secrete PEDF which maintains homeostasis within the retina, whilst PEDF is downregulated in eyes of patients with AMD and GA [2]. PEDF's multifaceted properties, including its ability to promote cell survival, reduce inflammation, inhibit angiogenesis, combat oxidative stress, regulate autophagy, and stimulate anti-apoptotic pathways, make it a promising therapeutic candidate for GA.

The accumulation of drusen is a hallmark of AMD and GA. In healthy eyes, drusen is recognised by scavenger receptors (e.g., CD36) on the RPE which trigger their removal by phagocytosis [3], thus preventing accumulation of debris in the subretinal space which can disrupt the retinal structure and function, prevent the delivery of nutrients to the photoreceptors, and decrease the ability of photoreceptors to respond to light. Although mechanisms by which PEDF enhances phagocytosis of drusen are not clear, deletion of PEDF has been shown to cause defective phagocytosis and senescent-like changes in the RPE [4], consequently limiting their retinoprotective capacity. Supplementation with PEDF may protect RPE cell function, thereby promoting the removal of drusen from the subretinal space and maintaining visual acuity.

Although high levels of reactive oxygen species (ROS) are an expected by-product of phototransduction and vision, the overproduction of ROS, also known as oxidative stress, is a significant contributor to retinal damage in GA [5]. The outer segments of photoreceptor cells are constantly exposed to light and are subject to oxidative stress due to their high metabolic activity. High levels of ROS can damage cell membranes through lipid peroxidation, inflammation, and DNA damage, and can affect protein integrity. In healthy eyes, the anti-oxidant properties of PEDF protect against oxidative stress, thereby maintaining cellular homeostasis and function of the tissue [6]. Therefore, supplementation with PEDF could, in theory, help maintain homeostasis in the highly metabolically active retinal tissue.

Apoptosis is a genetically regulated form of cell death that causes significant retinal cell loss in GA as a consequence of oxidative stress and inflammation [7]. PEDF has been found to activate anti-apoptotic pathways within retinal cells by triggering the expression of key anti-apoptotic proteins such as Bcl-2 that can inhibit the apoptotic cascade [6]. This action helps preserve the structural and functional integrity of the retina. Additionally, PEDF acts as a neurotrophic factor, providing essential growth and survival signals to photoreceptor cells, such as by stimulating the production of brain-derived neurotrophic factor (BDNF), a protein crucial for photoreceptor survival [8].

The conversion of GA to neovascular AMD (nAMD) is of significant concern since this can rapidly lead to legal blindness. The conversion to nAMD is driven by VEGF that causes the development of abnormal blood vessels within the retina. In its latent stage, the patient may not experience symptoms, however, these vessels often leak blood and impact RPE function leading to significant retinal damage and sight loss. GA is associated with meaningful disease burden and risk of nAMD as shown in a study of 18,712 eyes diagnosed with GA in which 8.8% of eyes developed nAMD in the first year [9]. Since PEDF is a potent endogenous inhibitor of the VEGF receptors (VEGFR-1 and VEGFR-2) [10], it is plausible that supplementation with PEDF could inhibit VEGF activity and thereby preserve the integrity of the retina.

PEDF is a multifunctional protein capable of protecting the RPE and supporting the critical relationship between photoreceptors and RPE cells. Since a number of clinical studies have shown PEDF levels are reduced in the eyes and blood of patients with AMD and GA [2][11][12], genetic supplementation with PEDF has the potential to restore function of the retina and prevent progression of the disease.

### Genetic delivery of PEDF

Although recombinant PEDF has been evaluated as a full-length protein or a functional polypeptide fragment in preclinical models of diabetic retinopathy [13] and nAMD [14], the relatively short half-life of recombinant PEDF after intravitreal injection is a major hurdle for long-term clinical treatment. Methods to extend the half-life of the PEDF protein including encapsulation in polyethylene glycol (PEG)-modified nanostructured carriers (NLC) [15], PLGA nanoparticles [13], and lipid nanoparticles [16] have been evaluated in preclinical models. Whilst these approaches demonstrate the advantage of controlled release and re-dosing potential, they are limited by complex manufacturing processes, lesser understood pharmacokinetics, and uncertain regulatory hurdles. Genetic delivery of the PEDF transgene has the potential to overcome the issue of the short half-life of the recombinant protein by enabling the genetically modified cells to produce a long-lasting, stable therapeutic expression following a single injection to the eye. The eye is a particularly good target for gene therapy due to its relatively small size, physical accessibility, and ease of administration.

Gene therapies can be delivered in a number of different ways, including viral vectors, nonviral vectors, and physical methods such as electroporation. Each of these approaches have their own advantages and limitations, therefore when choosing the optimal delivery vehicle, it is important to consider the packaging capacity, genome integration, duration of expression, target specificity and safety. With respect to genetic delivery to the eye, currently Adeno-associated virus (AAV) vectors are the most popular vector for the development of ocular gene therapies due to their lack of pathogenicity, relatively low immunogenicity, ability to transduce non-dividing retinal cells and maintenance of sustained levels of expression following a single administration. There are several approved AAV gene therapies in the United States and Europe including Luxturna that is used to treat a genetic eye condition called Leber congenital amaurosis [17]. While AAVs offer numerous advantages, they are limited on the size of their cargo (~4.7kb). However, at around 50kDa (approximately 1.44kb), PEDF would comfortably sit within this packaging limit with added capacity for additional enhancers.

Lentiviral vectors have a relatively large packaging capacity compared to AAVs and are capable of transducing and integrating into non-dividing cells leading to stable expression. Lentiviral vectors expressing PEDF have been evaluated in preclinical ocular angiogenic models following intravitreal [18] or subretinal administration, with evidence of enhanced anti-angiogenic properties when combined with VEGF-miRNA [19]. Adenovirus-based gene therapy vectors efficiently transduce multiple ocular cell types with rapid onset following intravitreal delivery. An adenovirus vector designed to express PEDF showed inhibition of choroidal neovascularisation and a reduction in lesion size in preclinical models of AMD [20] and in clinical trials [21] respectively. However, despite adenovirus- and lentivirus-based gene therapies being well tolerated following clinical administration to the eye [22], there are several hurdles to overcome including short-term expression for the use of adenovirus vectors and limitations in specific retinal cell targeting for the lentiviral vectors and currently most viral gene therapies under active development for ocular disorders use AAVs as the vector of choice.

Nonviral genetic delivery methods include lipid nanoparticle (LNP) delivery of mRNA [23], and electrotransfection of DNA plasmids [24], amongst others. LNPs are advantageous as they theoretically allow for precise targeting of specific cells within the retina, which is essential for intracellular or membrane proteins, but this is less important for secreted factors, such as PEDF, and involves complex manufacturing processes. Immunogenicity and long-term safety profiles of LNPs are not currently well understood. Electrotransfection targets the ocular ciliary muscles and therefore could secrete proteins like PEDF into the eye. Not requiring a viral packaging step, this approach is significantly less affected by cargo size and benefits from reduced manufacturing complexity. However, long-term efficacy of this approach has not been demonstrated, and it remains unclear how multiple administrations will affect the muscle tissue, especially in aged, GA eyes.

### Critical challenges and Future Directions

Although there are a multitude of potential advantages associated with genetically delivering PEDF for neuroprotection in GA, there remain several obstacles requiring further investigation. The primary challenge is the transition from preclinical studies to clinical trials. Whilst preclinical studies can demonstrate the mechanism of action and the potential benefits of a PEDF-based therapy, they do not necessarily predict real-world effects. There is also the additional challenge of identifying the GA patient population who will benefit the most from therapeutic administration of PEDF; intervention at the stage of nascent GA may help prevent or slow the development of GA, whereas treatment of eyes with advanced GA could slow further disease progression and reduce the likelihood of nAMD conversion.

Although genetic delivery of PEDF overcomes the issue related to stability and the need for regular dosing, there remains the challenge of the optimal delivery method and capacity to reach the disease-specific target tissues within the eye. If PEDF levels diminish too quickly or become excessively high, it could lead to suboptimal treatment outcomes.

In preclinical PEDF-based studies intravitreal injection is a commonly used delivery route due to the relative ease of administration. However, clinically intravitreal injection carries a risk of infection and inflammation and in eyes with GA, where the ocular environment is already inflamed, this could further harm retinal tissues and reduce the efficiency of gene transfer. Intravitreal injection is generally less efficient for targeting cells in the outer retina due to dilution of the vector in the vitreous cavity and having to traverse the many retinal layers. Subretinal and suprachoroidal techniques are attractive because they target RPE cells in the outer retina, thereby reducing off-target effects of transducing non-retinal cells and minimising the risk of adverse inflammatory reactions [22]. However, subretinal delivery requires specialised skills to prevent the development of a retinal detachment or macular hole. In contrast, the suprachoroidal route combines the relative ease of the intravitreal injection with the outer retinal targeting of the subretinal approach and is fast becoming a popular ocular delivery route [25,26].

To date, the safety of genetically delivered PEDF has exhibited a favourable safety profile, with a relatively low incidence of therapy-related adverse events as illustrated in the Ad.PEDF clinical trial in eyes with nAMD [21]. Since PEDF is a naturally occurring protein that is normally expressed by the RPE, it is not expected to be immunogenic if delivered genetically to the eye. Nevertheless, it is crucial to consider immunomodulatory strategies and maintain continuous patient monitoring for those receiving viral or non-viral-based treatments to promptly detect and manage any adverse immune reactions. Ongoing research and innovation in this field remain essential for unlocking new therapeutic avenues to decelerate degenerative processes and ultimately preserve or enhance visual function in GA patients.

GA involves complex pathological pathways, encompassing oxidative stress, inflammation, dysregulation of the complement system, and vascular abnormalities [5]. Combining PEDF with other components that target different aspects of these pathways may offer potential synergy, and a more effective treatment strategy. One intriguing consideration is the combination of PEDF with anti-complement therapies, which could more effectively address the inflammatory aspect of the disease while providing support for RPE cell survival and function, critical for maintaining retinal health. Together, a PEDF and anti-complement could offer a more holistic approach to slowing the rate of GA progression, and this approach has shown promise in pre-clinical models of GA [27]. The development of combination therapies requires careful consideration, therefore thorough preclinical and clinical research is necessary to comprehensively evaluate the safety profiles and optimal efficacy dosages. The synergistic potential of combining PEDF with other therapeutic components, as seen in combinational therapies for glaucoma [28] and nAMD [29], presents an exciting avenue for enhancing GA management. Such combinations may not only improve therapeutic outcomes but also provide a more comprehensive and targeted approach to addressing this challenging retinal disease.

### Conclusion

PEDF represents a promising therapeutic strategy for addressing a form of advanced AMD with limited treatment options. PEDF's multifunctional properties, including neuroprotection, anti-angiogenesis, and anti-inflammation, make it an attractive candidate for preserving visual function in GA patients. Ongoing research, optimization of delivery methods, and clinical trial design will be crucial in realising the full therapeutic potential of PEDF for GA, potentially offering hope to those suffering from this debilitating retinal condition.
